# Activation and cleavage of SASH1 by caspase-3 mediates an apoptotic response

**DOI:** 10.1038/cddis.2016.364

**Published:** 2016-11-10

**Authors:** Joshua T Burgess, Emma Bolderson, Mark N Adams, Anne-Marie Baird, Shu-Dong Zhang, Kathy A Gately, Kazuo Umezawa, Kenneth J O'Byrne, Derek J Richard

**Affiliations:** 1 Cancer and Ageing Program, Institute of Health and Biomedical Innovation at the Translational Research Institute (TRI), Queensland University of Technology (QUT) and Princess Alexandra Hospital, Level 6, Translational Research Institute, Brisbane, QLD, Australia; 2 Princess Alexandra Hospital, Ipswich Road, Woolloongabba, Brisbane, QLD 4102, Australia; 3 Northern Ireland Centre for Stratified Medicine, University of Ulster, C-TRIC Building, Altnagelvin Hospital Campus, Glenshane Road, Londonderry BT47 6SB, UK; 4 Center for Cancer Research and Cell Biology, Queen's University Belfast, Belfast, UK; 5 Thoracic Oncology Research Group, Institute of Molecular Medicine, Trinity College Dublin, St. James's Hospital, Dublin, Republic of Ireland; 6 Department of Molecular Target Medicine Screening, Aichi Medical University, Nagakute, Japan

## Abstract

Apoptosis is a highly regulated cellular process that functions to remove undesired cells from multicellular organisms. This pathway is often disrupted in cancer, providing tumours with a mechanism to avoid cell death and promote growth and survival. The putative tumour suppressor, SASH1 (SAM and SH3 domain containing protein 1), has been previously implicated in the regulation of apoptosis; however, the molecular role of SASH1 in this process is still unclear. In this study, we demonstrate that SASH1 is cleaved by caspase-3 following UVC-induced apoptosis. Proteolysis of SASH1 enables the C-terminal fragment to translocate from the cytoplasm to the nucleus where it associates with chromatin. The overexpression of wild-type SASH1 or a cleaved form of SASH1 representing amino acids 231–1247 leads to an increase in apoptosis. Conversely, mutation of the SASH1 cleavage site inhibits nuclear translocation and prevents the initiation of apoptosis. SASH1 cleavage is also required for the efficient translocation of the transcription factor nuclear factor-*κ*B (NF-*κ*B) to the nucleus. The use of the NF-*κ*B inhibitor DHMEQ demonstrated that the effect of SASH1 on apoptosis was dependent on NF-*κ*B, indicating a codependence between SASH1 and NF-*κ*B for this process.

Apoptosis is an essential cellular program designed to remove unwanted cells from organs and tissues. Apoptosis is also a crucial process for normal human embryonic development. Apoptotic pathways can be initiated by several mechanisms including the induction of excessive genome instability. Once activated apoptosis results in a coordinated and controlled process that ultimately leads to cell death. Disruption of the apoptotic process contributes to the development of many human diseases including cancer and immune disorders.^1, 2^ Key modulators of the apoptotic response are the caspase family of cysteine proteases. Caspase-9 activation is an early apoptotic event occurring after the release of cytochrome *C* from the mitochondria. Once activated caspase-9 cleaves downstream caspases resulting in the progression of the apoptotic response.^3^ Of this caspase family, caspase-3, caspase-6 and caspase-7 are the major effector proteases in apoptosis.^3, 4^ The proteolytic activity of the caspase family is tightly regulated, and upon activation, these proteases cleave numerous substrates at specific sites. In general, caspase substrates become inactivated upon cleavage; however, a subset become activated and contribute to apoptosis.^5^ To understand completely the role of caspases in apoptosis, it is essential to identify their downstream targets.

SAM and SH3 domain containing 1 (*SASH1*) is a putative tumour suppressor gene. In breast, lung, thyroid and colorectal cancers, SASH1 mRNA levels were found to be significantly reduced, compared with adjacent normal tissue.^6, 7^ Low SASH1 levels also correlate with aggressive tumour growth, metastasis and poor prognosis.^8, 9^ Methylation of the *SASH1* promoter (particularly CpG_26.27 and CpG_54.55) correlates with *SASH1* repression in breast cancer.^10^ The exact functions of SASH1 in normal tissues and in cancer are still unclear, but it is known to be localised to the nucleus and its SAM and SH3 domains imply signalling, adaptor and/or molecular scaffold functions.^11, 12^ The association of SASH1 with apoptosis has been reported in several studies.^7, 13, 14, 15^ For example, SASH1 depletion has been described to increase significantly cellular viability, proliferation and migration in A549 cells, whereas overexpression of SASH1 resulted in a significant increase in apoptosis.^7^ SASH1 overexpression has also been shown to affect apoptotic proteins including an increase in caspase-3 expression.^13^ Given the link of SASH1 with cancer, it is important to characterise the role of SASH1 in apoptosis to use SASH1 as a biomarker or therapeutic target.

NF-*κ*B has been described to both inhibit and promote apoptosis. NF-*κ*B is a transcription factor composed of five members: RelA (p65), RelB, c-Rel, NF-*κ*B1 (p105) and NF-*κ*B2 (p100), all of which contain a rel homology domain that is responsible for their DNA-binding and transcription regulatory functions.^16, 17^ The NF-*κ*B pathway is involved in diverse pathways including regulation of cell differentiation, proliferation and survival, as well as inflammatory cytokines.^18, 19, 20^ With such varied functions, the NF-*κ*B pathway is under tight regulation with positive and negative regulatory elements.^21^ NF-*κ*B is regulated by binding to the IkB protein, with this sequestering NF-*κ*B to the cytoplasm. Activation of NF-*κ*B occurs when IkB is phosphorylated by the IkB kinase, targeting IkB for proteasome degradation. This releases NF-*κ*B, allowing it to translocate to the nucleus.^22^ The NF-*κ*B-associated inhibitors of apoptosis c-IAP1 (cellular inhibitor of apoptosis protein-1), c-IAP2 and XIAP (X-linked inhibitor of apoptosis protein) suppress apoptosis through direct inhibition of effector caspases, whereas the members of the B-cell CLL/lymphoma 2 (bcl-2) family activate the proapoptotic members.^23, 24, 25^ Bcl-2 functions within cell survival by controlling the mitochondrial membrane permeability through the inhibition of the proapoptotic factors, BAX (BCL2-associated X protein) and BAK (BCL2 antagonist/killer 1). The increase in BAX and BAK from accumulative cellular stress as well as a decrease in bcl-2 levels results in mitochondrial membrane permeability releasing cytochrome *c* and activating APAF1 (apoptotic protease-activating factor 1)-induced caspase-9 cleavage and the apoptotic response.

In this study, we further characterise the mechanistic role of SASH1 in apoptosis. We demonstrate that depletion of SASH1 by siRNA results in resistance to UVC-induced apoptosis. Furthermore, we show that following induction of apoptosis, cytoplasmic SASH1 is cleaved by caspase-3 and this cleaved C-terminal fragment of SASH1 is translocated to the nucleus. Loss of this site prevents caspase-3 cleavage and results in the loss of nuclear SASH1. Further, we show that SASH1-mediated induction of apoptosis occurs through an NF-*κ*B-dependent mechanism. Interestingly, NF-*κ*B translocation to the nucleus following the initiation of apoptosis is at least in part dependent on SASH1.

## Results

**Figure 1:**
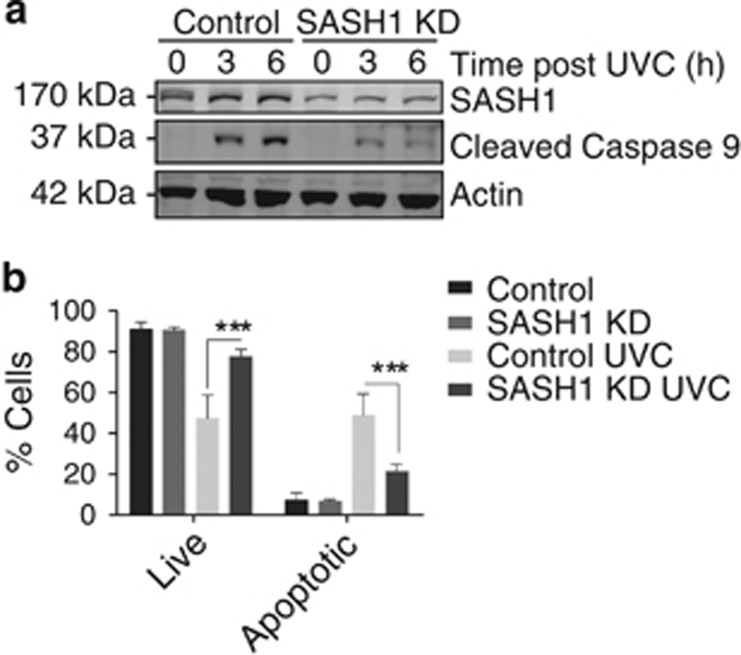
SASH1-depleted cells are resistant to apoptosis. (**a**) Immunoblot of control or SASH1-depleted (knockdown (KD)) HeLa cells 3 or 6 h following ultraviolet light C (UVC) exposure (30 mJ/cm^2^) probed for the apoptotic marker cleaved caspase-9 and *β*-actin as a loading control. Less cleaved caspase-9 was observed in SASH1-depleted cells, indicating a reduced apoptotic response to UVC. (**b**) Quantification of Annexin PI profiles of HeLa cells, control or KD. Cells were harvested (3 h after 30 mJ/cm^2^) and stained with Annexin V 488 and PI and analysed on the Gallios flow cytometer. Data shown are the means±S.D. from five independent experiments. Statistical analysis was performed with Student's *T*-test with ***P<0.0001

SASH1 has previously been reported to participate in the regulation of apoptosis.^7, 13, 14, 15^ To explore the molecular mechanism through which SASH1 functions in apoptosis, we initially depleted SASH1 in HeLa cells using esiRNA (endoribonuclease-prepared siRNA) and then irradiated these cells with 30 mJ/cm^2^ UVC to induce apoptosis through a caspase-3-dependent pathway. Cell lysates were analysed by immunoblot for caspase-9 activation via its autocleavage. This demonstrated that depletion of SASH1 reduced caspase-9 cleavage, suggesting that SASH1 has a role in the progression of apoptotic pathways (Figure 1a). To further explore if apoptosis had been suppressed in the absence of SASH1, we depleted SASH1 from HeLa cells using esiRNA and analysed the presence of surface-exposed Annexin V via FACS. Following UVC treatment, there were significantly less Annexin V-positive cells present in the SASH1-depleted cells as compared with the mock-treated cells, indicating that apoptosis was suppressed in the absence of SASH1 (Figure 1b). Consistently, depletion of SASH1 from A549 lung cancer cells (Supplementary Figure 1A) suppressed apoptosis (Supplementary Figure 1B), whereas the use of an alternative siRNA targeting SASH1 (GenePharma, Shanghai, China; Supplementary Figure 2A) also significantly suppressed apoptosis (Supplementary Figure 2B). The treatment of SASH1-depleted cells with staurosporine (Supplementary Figure 3A), which induces apoptosis by inhibiting protein kinase activity, demonstrated that the SASH1-depleted cells were more resistant to the staurosporine apoptotic stimuli.

**Figure 2:**
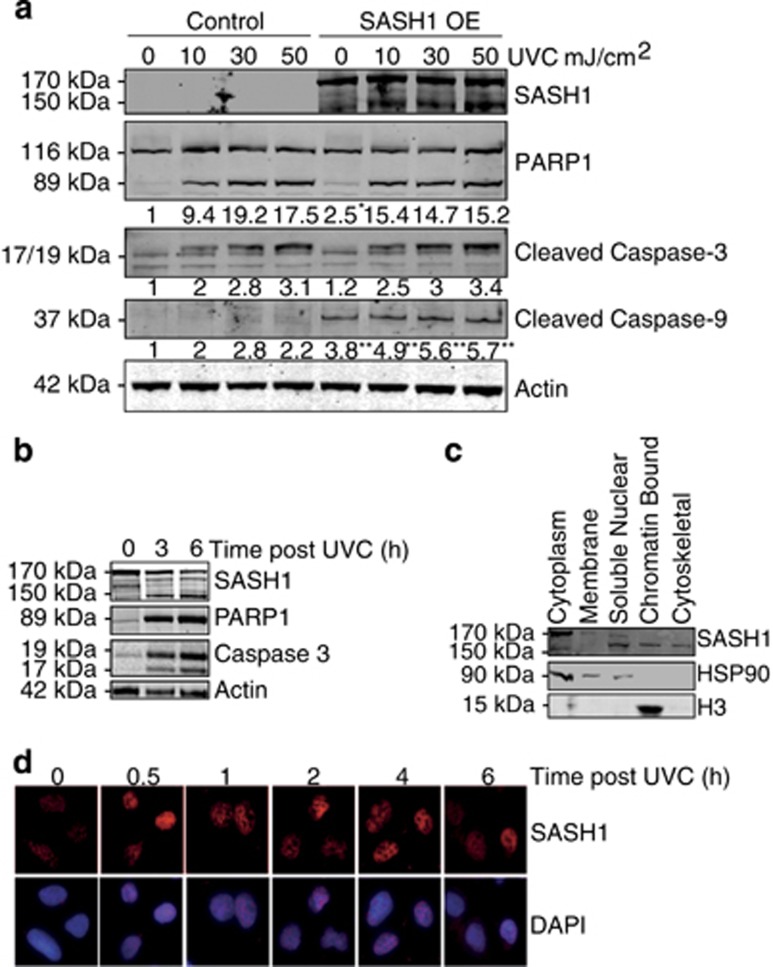
SASH1 overexpression induces apoptosis. (**a**) HeLa cells were transfected with Flag-SASH1 (OE=overexpressed) and irradiated with ultraviolet light C (UVC) 3 h to induce apoptosis (10–50 mJ/cm^2^). Quantification of PARP1, cleaved caspase-3 and cleaved caspase-9 levels indicated below blots wasperformed with ImageJ (University of Wisconsin, Madison, USA). Statistical analysis was performed with Student's *T*-test with *P<0.01 and **P<0.001. (**b**) Immunoblot of UVC-treated HeLa cells (3 h after 30 mJ/cm^2^) indicating cleavage of SASH1 from 170 to 150 kDa. PARP1, caspase-3 and caspase-9 antibodies were also used as markers of apoptosis. (**c**) HeLa cells were subjected to subcellular fractionation and immunoblotted. The immunoblot was incubated with SASH1, histone H3 as a chromatin marker and heat-shock protein 90 (HSP90) antibodies as a cytoplasmic marker (ThermoScientific Subcellular Fractionation Kit). (**d**) Representative images from immunofluorescence using SASH1 antibodies in HeLa cells following UVC (50 mJ/cm^2^). DAPI (4',6-diamidino-2-phenylindole) was used to stain the nucleus of the cells

The overexpression of SASH1 has been described to induce apoptosis.^7, 13, 14, 15^ To confirm this, we ectopically overexpressed SASH1 in HeLa cells and measured the cleavage of poly (ADP-ribose) polymerase (PARP1), caspase-3 and caspase-9 by immunoblot. This showed that overexpression of SASH1 increased the cleavage of proteins involved in apoptotic pathways before and after UVC treatment. This confirmed the observations from previous studies that SASH1 overexpression induces apoptosis (Figure 2a). While overexpression of SASH1 in A549 cells did not affect PARP1 cleavage, overexpression induced a marked increase in caspase-9 cleavage (Supplementary Figure 4A). Overexpression of SASH1 significantly increased apoptosis in A549 cells (Supplementary Figure 4B).

Interestingly, following induction of apoptosis in HeLa cells via UVC exposure or treatment with the staurosporine, a lower molecular weight antigenic band was observed on an immunoblot incubated with SASH1 antibodies (Figure 2b and Supplementary Figure 5). While the predicted molecular weight of full-length SASH1 is 170 kDa, the UVC-induced smaller SASH1 product was observed at ~150 kDa. To further investigate whether the 150 kDa SASH1 band is functional, we performed subcellular fractionation of HeLa cells. Immunoblotting demonstrated that while the 170 kDa full-length SASH1 was predominantly cytoplasmic, the 150 kDa form was predominantly nuclear (Figure 2c). Sigma HPA029947 anti-SASH1 antibody is raised against amino acid (744–838) located approximately in the middle of the SASH1 sequence; therefore, the detection of SASH1 following small cleavage events is possible. We next examined if the localisation of SASH1 to the nucleus was stimulated by UVC. HeLa cells were irradiated with UVC and fixed at time points after treatment. Cells were pre-extracted with a detergent buffer before fixation to remove non-chromatin-bound proteins,^26, 27^ and this consistent with our subcellular fractionations indicated that SASH1 localised to detergent-resistant chromatin structures following UVC irradiation (Figure 2d). Lower bands are also detected with the SASH1 antibody as well as the Flag antibody, which may indicate alternative cleavage events of SASH1 (Supplementary Figure 6A).

**Figure 3:**
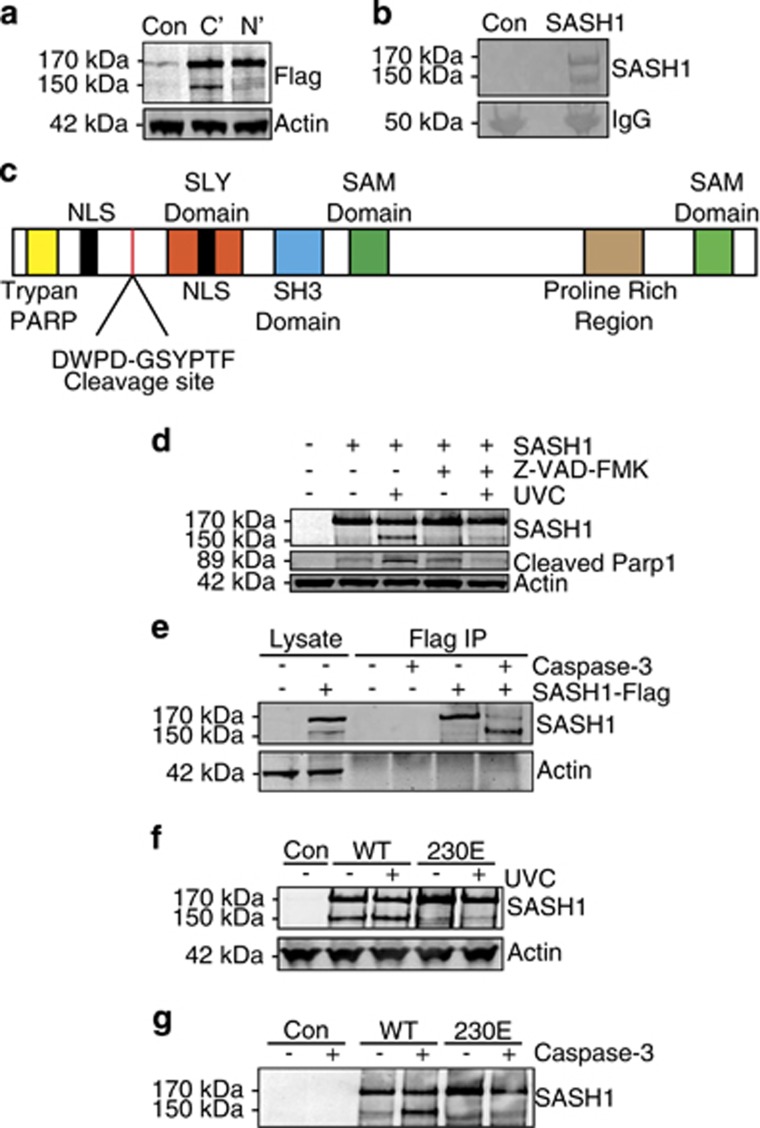
SASH1 is cleaved by caspase-3 at D230. (**a**) Immunoblot of U2OS cells overexpressing N- or C-terminal Flag-tagged SASH1. (**b**) Coomassie-stained sodium dodecyl sulfate-polyacrylamide gel electrophoresis (SDS-PAGE) gel containing immunoprecipitation of C-terminal Flag-tagged SASH1. (**c**) Schematic diagram of SASH1 protein domains indicating cleavage site and N-terminal sequencing of amino acids (GSYPTF), which are preceded by a caspase-3 recognition site (DXXD). (**d**) SASH1 was immunoprecipitated from SASH1-Flag stably expressing U2OS cells using M2 Flag beads, followed by induction of apoptosis by UVC (50 mJ/cm^2^, 3 h) with cells pre-treated (30 min) with caspase-3 inhibitor Z-VAD-FMK (20 *μ*M). (**e**) SASH1 is cleaved by recombinant caspase-3. SASH1 was immunoprecipitated from U2OS cells as per (**d**) and incubated with recombinant caspase-3 (2 U, 16 h). (**f**) Ectopically expressed 230E mutant SASH1 is not cleaved following UVC exposure. HeLa cells overexpressing wild-type or D230E were treated with UVC (50 mJ/cm^2^, 3 h). Cell extracts were immunoblotted and incubated with the indicated antibodies. (**g**) SASH1 230E mutant is not cleaved by recombinant caspase-3. Cell extracts taken from HeLa cells overexpressing wild-type and 230E SASH1 were incubated with recombinant caspase-3 (2 U, 16 h)

To explore if the 150 kDa form of SASH1 is the result of a proteolytic cleavage event, we ectopically expressed SASH1 in U2OS cells either with a N- or C-terminal flag tag fusion without additional apoptotic stimuli. Cell lysates were analysed by immunoblot using an anti-flag antibody. The 150 kDa form of SASH1 was not produced from the N-terminal-tagged SASH1, whereas a prominent 150 kDa band could be observed in the lysates from cells expressing C-terminal-tagged SASH1 (Figure 3a). This indicated that the 150 kDa lower molecular weight fragment of SASH1 was produced as the result of a cleavage event proximal to the N terminus.

To identify the exact site of SASH1 cleavage, we performed N-terminal sequencing. C-terminal Flag-tagged SASH1 was ectopically expressed in HeLa cells. The expressed protein was immunoprecipitated from cell lysates using M2 resin (Sigma-Aldrich, Castle Hill, NSW, Australia), transferred onto polyvinylidene fluoride (PVDF) and stained with Coomassie blue G250 (Figure 3b). The peptide corresponding to the cleaved N-terminal fragment of SASH1 was then excised and subjected to N-terminal sequencing. The cleavage site was identified as D*G corresponding to amino acids 230 and 231 (Figure 3c). This represented a putative caspase-3 recognition site (DWPD) and was located between the trypan PARP and SLY domains of SASH1 (Figure 3c). To confirm whether SASH1 is cleaved by caspase-dependent proteolysis, we exposed U2OS cells stably overexpressing SASH1 to UVC and treated the cells with or without the pancaspase inhibitor Z-VAD-FMK and immunoblotted for SASH1. Consistent with the involvement of a caspase protease, the 150 kDa form of SASH1 was not detected in lysates from cells exposed to UVC and treated with Z-VAD-FMK, suggesting that caspase inhibition prevents SASH1 cleavage (Figure 3d). To determine if SASH1 is cleaved by caspase-3 and not a downstream protease, we incubated immunoprecipitated (C-terminal Flag tag) SASH1 with recombinant purified caspase-3. Immunoblot analysis of the reaction indicated the presence of the cleaved 150 kDa SASH1 band after incubation with recombinant caspase-3 (Figure 3e). This revealed that SASH1 could be cleaved by caspase-3 in a purified reconstituted assay. Further, the overexpression of the C-terminal Flag-tagged SASH1 D230E mutant in HeLa cells inhibited the cleavage of SASH1 following UVC irradiation, confirming that the aspartic acid at amino acid 230 is essential for the caspase-3 cleavage (Figure 3f). Taken together, this supports the possibility that the cleavage of this site is by a caspase protease. To confirm the site of cleavage, we next incubated immunoprecipitated wild-type SASH1 and the SASH1 caspase-3 cleavage site mutant D230E with recombinant caspase-3. Immunoblot analysis indicated that the D230E mutant protein could not be cleaved by recombinant caspase-3, confirming that the site of cleavage is D230 (Figure 3g).

**Figure 4:**
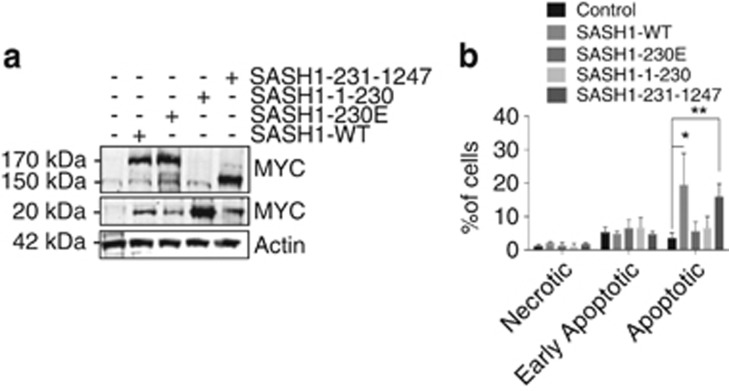
Overexpression of SASH1 amino acids 231–1247 induces apoptosis. (**a**) Immunoblot indicating the overexpression of SASH1 fragments from the cells used in (**b**). (**b**) Annexin V and PI staining of HeLa cells 48 h following overexpression of control (empty PCMV6 vector), SASH1 WT, SASH1 230E, SASH1 1–230 or SASH1 231–1247 fragments. Fluorescence of cells was measured using a Gallios flow cytometer (Beckman) and was quantified using the Kaluza software (Beckman). The percentage of live and apoptotic cells is shown. The data represent the average and S.D. of three independent experiments. Unpaired *T-*test with *P<0.05 and **P<0.005

We next sought to determine if the cleavage of SASH1 is required for the apoptotic function of SASH1. To explore this possibility, the SASH1 N- and C-terminal fragments were cloned and ectopically expressed in HeLa cells (Figure 4a). Transduced HeLa cells were analysed for necrosis, early apoptosis and late apoptosis in the absence of UVC. This indicated that overexpression of SASH1 in HeLa cells increased baseline apoptosis as expected, whereas overexpression of the uncleavable D230E mutant showed no increase in apoptosis (Figure 4b). Overexpression of the C-terminal SASH1 fragment was observed to act in the same manner as the full-length SASH1, suggesting that this fragment (231–1247) was enough to prime cells for apoptosis (Figure 4b). Interestingly, these data demonstrate that overexpression of SASH1 or the 231–1247 fragment of SASH1 initiates apoptosis in cells in the absence of an additional apoptotic stimulus.

**Figure 5:**
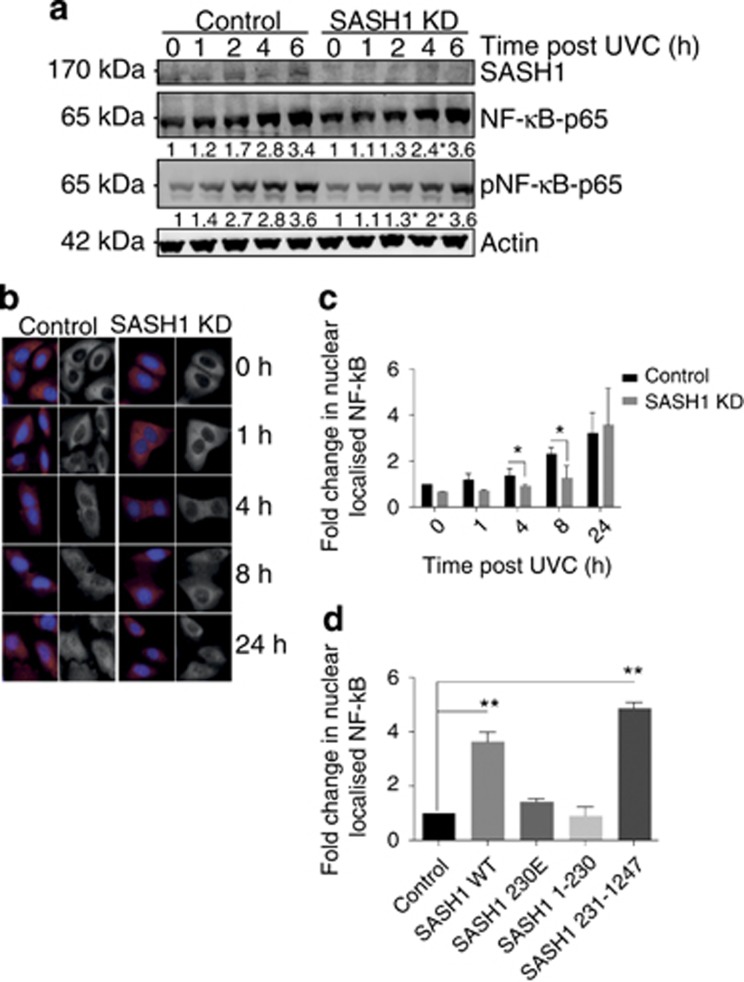
SASH1 is required for NF-*κ*B localisation to the nucleus. (**a**) SASH1 is required for NF-*κ*B stabilisation and phosphorylation. Immunoblot of SASH1-depleted cells following UVC treatment. HeLa cells were transfected with control or SASH1 small interfering RNA (siRNA) 48 h before treatment with 30 mJ/cm^2^ UVC. Cell extracts were isolated at the time indicated after UVC treatment, immunoblotted and incubated with the indicated antibodies. (**b**) Nuclear localisation of NF-*κ*B following UVC induction with or without SASH1 depletion. HeLa cells were treated as in (**a**) and fixed at the indicated timepoints. Immunofluorescence was performed using NF-*κ*B p65 antibodies. (**c**) Quantification and statistical analysis was performed of (**b**) with InCell 2200 and InCell analysis software. The data represent the average and standard deviation of three independent experiments. Unpaired *T*-test with *P<0.05. (**d**) Overexpression of SASH1 WT or 231–1247 increases NF-*κ*B nuclear levels. Quantification of HeLa cells from fixed cells imaged with Incell 2200 and analysed with Incell analyser software. The data represent the average and standard deviation of three independent experiments. Unpaired *T*-test with **P<0.005

Previous studies have shown a role for SASH1 in regulating NF-*κ*B activity.^28^ SASH1 was shown to independently bind I*κ*B kinase *α* and I*κ*B kinase *β*, which resulted in the promotion of NF-*κ*B signalling. NF-*κ*B also has a role in apoptosis, functioning to regulate the expression of anti- and proapoptotic factors.^21, 24^ In light of the above, we next sought to determine whether SASH1 influenced apoptosis through an NF-*κ*B-dependent mechanism. HeLa cells were depleted of SASH1 or mock treated and irradiated with UVC to induce apoptosis. While normal NF-*κ*B phosphorylation and accumulation was observed in the control siRNA-treated cells, there was a delay in NF-*κ*B activation in SASH1-depleted cells, suggesting that SASH1 is required for efficient activation of NF-*κ*B (Figure 5a). After induction of apoptosis NF-*κ*B is translocated to the nucleus. We next investigated the nuclear translocation of NF-*κ*B in cells transfected with control or SASH1 siRNA via immunofluorescence. Depletion of SASH1 significantly disrupted the translocation of NF-*κ*B to the nucleus (Figure 5b), suggesting that SASH1 is required for NF-*κ*B recruitment to the nucleus to promote apoptosis. To explore if cleaved SASH1 was required for NF-*κ*B translocation, we expressed recombinant SASH1, D230E SASH1 and the SASH1 C-terminal fragment 231–1247 in HeLa cells exposed to UVC. It was observed that the expression of full-length SASH1 (WT) and 231–1247 SASH1 led to an increase in nuclear NF-*κ*B regardless of the presence of endogenous SASH1 protein. Therefore, NF-*κ*B activation was dependent on D230 of SASH1 and expression of the C-terminal fragment alone was enough to activate NF-*κ*B and stimulate its translocation to the nucleus (Figure 5c). The localisation of SASH1 to the nucleus was also shown to be dependent on SASH1 cleavage, with D230E having significantly less nuclear staining relative to cytoplasmic staining while the nuclear levels of 1–230 and 231–1247 SASH1 were elevated (Supplementary Figure 7A). In addition, SASH1 230E did not display a significant increase in SASH1 nuclear levels following UVC, in contrast to the increase in SASH1 WT or SASH1 mutant 231–1247 nuclear levels (Supplementary Figure 7B).

**Figure 6:**
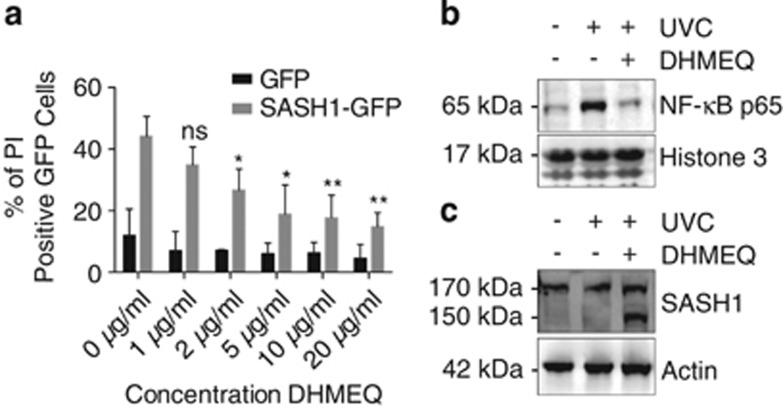
SASH1-induced apoptosis is dependent on the NF-*κ*B pathway. (**a**) HeLa cells overexpressing SASH1-GFP were treated with DHMEQ for 48 h after transfection and were live stained with PI (dead marker) and Hoechst (nuclear marker). Cells were imaged with an InCell 2200 and apoptotic GFP-positive cells were quantified. The data shown are the means±S.D. from three independent experiments. (**b**) Immunoblot of the chromatin fraction of HeLa cells following UVC (30 mJ/cm^2^, 3 h) with or without DHMEQ 10 *μ*g/ml. Confirming DHMEQ inhibits NF-*κ*B-p65 localisation to the nucleus following UVC stimuli. (**c**) Immunoblot of the cytoplasmic fraction of HeLa cells following UVC (30 mJ/cm^2^, 3 h) treated with or without DHMEQ (10 *μ*g/ml). Inhibition of NF-*κ*B-p65 induces an accumulation of SASH1 150 kDa band in the cytoplasm

Although NF-*κ*B required SASH1 for efficient activation and translocation to the nucleus, we next investigated whether the SASH1-induced apoptosis was dependent on NF-*κ*B. HeLa cells were ectopically transfected with empty vector GFP (green fluorescent protein) or SASH1-GFP and treated with escalating doses of the NF-*κ*B inhibitor DHMEQ.^29, 30^ Apoptosis induced by the overexpression of SASH1-GFP was significantly reduced in a dose-dependent manner by inhibition of NF-*κ*B activation (Figure 6a). Additionally, DHMEQ reduced the relative SASH1 wild-type and SASH1 mutant 231–1247 nuclear levels (Supplementary Figure 7C). Subcellular fractionation was performed to confirm that DHMEQ prevents NF-*κ*B P65 binding chromatin following UVC stimuli (Figure 6b). Furthermore, subcellular fractionation showed that the inhibition of NF-*κ*B with DHMEQ resulted in an accumulation of cleaved SASH1 in the cytoplasm, suggesting that NF-*κ*B activity is required for SASH1 nuclear localisation (Figure 6c). Next, we examined the expression of apoptotic genes, which are in part regulated by NF-*κ*B P65. There was a significant increase in the mRNA expression of antiapoptotic genes following SASH1 knockdown. In contrast, there was a significant decrease in the same antiapoptotic genes following SASH1 overexpression (Supplementary Figure 8). The proapoptotic gene *BAX* also showed a decrease following SASH1 knockdown; however, this did not reach statistical significance. These data suggest that the induction or inhibition of apoptosis in SASH1-overexpressing or -depleted cells, respectively, is through an NF-*κ*B-dependent mechanism.

## Discussion

Apoptosis is a critical cellular process in multicellular organisms enabling the controlled removal of unwanted cells. In cancer, SASH1 is reported to function as a putative tumour suppressor. For example, loss of SASH1 is associated with resistance to apoptosis, while overexpression of SASH1 leads to an induction of apoptosis.^7^ However, it remains unclear how SASH1 functions in apoptotic pathways. In this study, we examined the role of SASH1 in the activation of apoptosis in response to UVC radiation. We demonstrated that depletion of SASH1 prevented apoptosis in cells treated with UVC or staurosporine. Similar to previous studies,^7, 13, 14^ we also showed that overexpression of SASH1 led to an increase in apoptotic cells in both HeLa and A549 cell lines. We also characterised the cleavage of SASH1 and demonstrated that expression of the C-terminal cleavage fragment including amino acids 231–1247 was able to induce apoptosis in the absence of exogenous apoptotic stimuli. We lastly demonstrated that SASH1-associated apoptosis was driven through an NF-*κ*B-dependent pathway.

Following UVC or staurosporine treatment, a pool of SASH1 was observed to increase at a lower molecular weight. Interestingly, subcellular fractionation revealed that the nuclear SASH1 migrated at a lower molecular weight, suggesting that a cleavage event had occurred. Caspase-3, -6 and -7 are the three proteases that are responsible for the proteolysis of proteins for induction of apoptosis.^4, 31^ To identify the downstream events of apoptosis, it is of fundamental importance to identify the caspase substrates. Here we have demonstrated that the apoptosis regulator caspase-3 is responsible for SASH1 cleavage between amino acids 230 and 231. Many proteins have been shown to be cleaved by caspases during apoptosis including the multifunctional protein PARP1 and the human DNA repair nuclease Exo1.^32^ The cleavage of SASH1 was shown to be required for its apoptotic role, which indicates it functions downstream to the initiation of caspase-3 (therefore a modifier of apoptosis); however, as overexpression of SASH1 increases caspase-9 and initiates apoptosis, it could also function to some degree as an inducer. Clearly, multiple feedback loops occur within the apoptotic pathways, which SASH1 appears to be involved with. The cleavage of nuclear proteins is essential for apoptosis to proceed, such as the caspase-activated DNase, which is required for DNA fragmentation. In addition, PARP-1, a DNA repair-associated protein, is cleaved by caspases following apoptotic induction, resulting in the formation of both pro- and antiapoptotic PARP-1 fragments.^33, 34^ Caspase-6 has been shown to cleave lamins A and C, whereas acinus cleavage by caspase-3 is required for chromatin condensation.^35, 36^ Interestingly, cleavage of SASH1 removes the N-terminal part of SASH1 containing the trypan PARP domain, thought to be involved in membrane binding. Therefore, cleavage of SASH1 might disrupt its predicted membrane interaction as well as subsequent protein interactions, allowing its relocation to the nucleus.

We next aimed to determine the mechanism behind the role of SASH1 in apoptosis. A previous study has suggested that SASH1 has a role in regulating the transcription factor NF-*κ*B.^28^ SASH1 acts as a molecular scaffold to promote the ubiquitination of TRAF6 (TNF receptor-associated factor 6, E3 ubiquitin-protein ligase) and assemble a complex containing the NF-*κ*B cofactors TRAF6, TAK1 (transforming growth factor *β*-activated kinase 1), IKKa (I*κ*B kinase a) and IKKb, thereby facilitating activation of NF-*κ*B.^28^ Of relevance to the current study, NF-*κ*B also has a role in DNA damage-induced apoptosis and cells depleted of the p65 NF-*κ*B subunit are, like SASH1-depleted cells, resistant to UV-induced apoptosis.^21^ NF-*κ*B can be observed to translocate to the nucleus upon induction of apoptosis, where it activates specific promoters. In light of the above, we next examined the effect of overexpression of SASH1 on NF-*κ*B activation. Expression of wild-type and the C-terminal-cleaved fragment of SASH1 led to an increase in nuclear NF-*κ*B, correlating with the increased apoptosis in these cells. Expression of the N-terminal SASH1 fragment or the caspase-3-resistant SASH1 D230E did not increase the levels of nuclear NF-*κ*B. This suggests that the C-terminal fragment of SASH1 is required to promote NF-*κ*B translocation into the nucleus. Inversely, depletion of SASH1 disrupted the nuclear recruitment of NF-*κ*B following UVC treatment, supporting a role for SASH1 in NF-*κ*B proapoptotic activation and translocation to the nucleus.

Interestingly, while we have demonstrated that SASH1 is required for timely NF-*κ*B activation, the NF-*κ*B inhibitor, DHMEQ, prevented apoptosis in cells overexpressing SASH1 in a dose-dependent manner, suggesting that the mechanism of SASH1 induction of apoptosis is, at least in part, through an NF-*κ*B-dependent pathway. Therefore, we propose that the C-terminal fragment of SASH1 translocates into the nucleus and in turn promotes NF-*κ*B proapoptotic roles and reduces NF-*κ*B antiapoptotic roles. The control of NF-*κ*B translocation is driven through the degradation of the IKK subunits, and loss of these IKK subunits results in the activation of NF-*κ*B leading to nuclear translocation.^37^ SASH1 is reported to have an interaction with the IKK subunits *α* and *β* either directly or through a protein complex.^28^ Therefore, it is possible that caspase-3-mediated cleavage of SASH1 may act as another regulatory mechanism to control IKK-mediated translocation of NF-*κ*B into the nucleus to promote apoptosis.

Recent publications have described SASH1 as having an inhibitory role on cellular proliferation through the PI3K/Akt (phosphatidylinositol-4,5-bisphosphate 3-kinase/protein kinase B) pathways.^38, 39^ SASH1 overexpression was demonstrated to reduce the phosphorylated PI3K and Akt levels while additionally having effects on epithelial–mesenchymal transition through the increase of E-cadherin and reduction of N-cadherin protein levels. The interplay between NF-*κ*B and PI3K/Akt signalling has previously been described with Akt being shown to enhance NF-*κ*B activity through the degradation of IkB.^40^ The roles of SASH1 within the cellular proliferation pathways (PI3K/Akt) clearly have implications on the results described here. It is possible that the effect of SASH1 on NF-*κ*B levels may be, in part, through its role in the PI3K/Akt pathway, SASH1 potentially functioning as a decision point influencing the cells fate leading to cell survival, proliferation or apoptosis.

SASH1 has been shown to be downregulated in many cancers.^6^ These data are highly significant for cancer treatment as they suggest that cancer cells with low SASH1 levels might be more resistant to DNA damage-induced apoptosis than cancer cells with higher levels of SASH1. We have shown here that SASH1 contributes to apoptosis via an NF-*κ*B-dependent mechanism. Accordingly, drugs that target NF-*κ*B might be an effective strategy to target tumours with low SASH1 levels. Indeed, further investigation is required to determine if this strategy alone or in combination with DNA-damaging agents might prove useful in the clinic.

## Materials and Methods

### Cell culture

Cells were cultured at 37 °C with 5% CO_2_ in RPMI media and supplemented with 10% FCS. Cells were passaged with trypsin and maintained at low passages. Cells were obtained from ATCC (Manassas, USA).

### Western blot analysis

Immunoblotting was carried out as described previously.^41^ Cells were harvested via scraping in media to retain floating (dead cells) or trypsinisation from tissue culture plates and were resuspended in immunoprecipitation (IP) buffer (20 mM HEPES, pH 8, 150 mM KCl, 5% glycerol, 10 mM MgCl_2_, 0.5% IGEPAL, 0.5 mM EDTA, 0.5 mM dithiothreitol (DTT), protease inhibitor cocktail (Invitrogen, ThermoFisher Distributer; Brendale QLD, Australia) (1:100), phosphatase inhibitor cocktail (Invitrogen) (1:100)) and sonicated to lyse the cells (Vibra Cell, Sonics and Materials, 3 mm probe at amplitude 40 for 10 s). Lysates were then centrifuged at 14 000 × *g* to remove the cellular debris. Samples (20 *μ*g) were run on Bolt 4–12% gradient gels (165 V for 40–90 min) (Invitrogen) and proteins were transferred (35 V, 1 h) into a transfer buffer (Tris-Base 25 mM, glycine 80 mM, 0.15% SDS, 20% methanol) and then to a nitrocellulose membrane (GE HealthCare Lifesciences, Parramatta NSW, Australia). Membranes were blocked in 2% fish skin gelatin, 0.1% Tween-20 in PBS (Sigma) (1 h) and incubated with primary antibody at 4 °C (overnight) in the same buffer. Following washing, fluorescently labelled secondary antibodies were then incubated with the blot in 2% fish skin gelatin and 0.1% Tween-20 in PBS (Sigma) (1 h) as indicated. Washed membranes were visualised using the Li-COR Odyssey infrared scanner (Mulgrave, VICTORIA, Australia).

### SASH1 knockdown

Depletion of SASH1 was performed using Sigma esiRNA or GenePharma siRNA. Transfections at a final concentration of 20 nM esiRNA or 50 nM GenePharma siRNA with RNAimax (Life Technologies, ThermoFisher Distributer; Brendale QLD, Australia) were performed as per the manufacturer's instructions. Double transfections were used to achieve optimal knockdown, where the second transfection was performed 24 h after the initial round of transfection. Optimal knockdown of SASH1 was observed at 72 h after initial transfection. GenePharma siRNA sequence 5′-GCAGCAGUAUGCAGAUUAUTT-3′.

### Immunofluorescence microscopy

Immunofluorescence was performed as described previously.^32, 42^ Briefly, soluble proteins were extracted using extraction buffer to enrich the chromatin-bound nuclear protein signal (20 mM HEPES (pH 8), 20 mM NaCl, 0.1 mM NaF, 0.5% Igepal) for 5 min, the cells were then fixed with 4% paraformaldehyde in PBS for 20 min and cells were left in PBS at 4 °C until stained. For immunostaining, cells were first permeabilised in 0.2% Triton X-100 for 5 min and then blocked (3% BSA in PBS) for 1 h, followed by incubation with primary antibodies as indicated. After incubation, cells were washed three times with PBS before the addition of secondary antibodies as indicated, and these were incubated for 1 h at room temperature (Alexa Fluro 594 with a 1:400 dilution (Life Technologies) in 3% BSA). Nuclear DNA was stained with DAPI (Sigma) for 5 min at 1 *μ*g/ml. A Delta Vision Elite Live Imaging Microscope (Applied Precision, GE HealthCare Lifesciences, Parramatta NSW, Australia) and softWoRx analysis software (Applied Precision) were used for the cell imaging. High-throughput analysis was performed using a Incell 2200 (GE Healthcare Lifesciences, Parramatta NSW, Australia) and a Incell investigator analysis software (GE Healthcare).

### SASH1 transduction

Myc-tagged *SASH1* constructs were cloned into pLEX 307 via a LR reaction. HEK293T virus-producing cells were cultured in DMEM containing 10% FCS at low passage. A T75 flask of cells was transfected with virus component plasmids (15 *μ*g of pLP1, 6 *μ*g of pLP2, 3 *μ*g of pVSV-G) with Lipofectamine 2000 (ThermoFisher; Brendale QLD, Australia). Virus containing media were collected 24 and 48 h after transfection. Cellular debris was removed by centrifugation at 600 × *g* for 10 min. Virus was used fresh or stored at −80 °C. Transduction of HeLa cells was performed by the addition of virus-containing medium to cells. Polybrene 1:6000 (Sigma-Aldrich) was used to increase transduction efficiency, with a second transduction performed 6 h after the first transduction. Cells were left 48 h after initial transduction before being harvested for experiments. SASH1 fragment overexpression was assessed by western blot analysis.

### SASH1 overexpression

The *SASH1* gene was cloned into a mammalian expression vector PCMV6 (Origene, Dianostic Technology; Belrose NSW, Australia). For a T25 flask, 3 *μ*g of DNA and 6 *μ*l of Lipofectamine 2000 was used, with DNA and Lipofectamine incubated separately for 5 min and then combined and allowed to incubate for 20 min before being added to the cells as per the manufacturer's instructions. Cells were harvested 24–48 h after transfection.

### Annexin V/PI analysis

HeLa or A549 cells transduced with SASH1, were trypsinised and stained as per the instructions of Promega Annexin V-FITC Apoptosis Detection Kit (United Bioresearch; Dural NSW, Australia). Cells were resuspended to 1 × 10^6^–2 × 10^6^ cells per ml in a 1 × binding buffer with 1 : 40 dilution of 488-Annexin antibody and then incubated for 20 min in the dark. Cells were washed in a 1 × binding buffer and then resuspended in 1 × binding buffer containing 1 : 20 dilution of propidium iodide (PI). Fluorescence of cells was measured using the Gallios flow cytometer system and was analysed using the Kaluza software (Beckman, Lane Cove NSW, Australia).

### N-terminal sequencing

Ectopically expressed SASH1 was immunoprecipitated from 4 × T175 flasks. The immunoprecipitation sample was then electrophoresed into a Bolt gel 4–12% (Invitrogen) and immunoblotted onto the PVDF membrane. The membrane was then stained with Coomassie R250 to visualise SASH1. The band representing the smaller cleaved fragment of SASH1 was excised from the blot using a sterile scalpel and sent to APAF for N-terminal sequencing with six rounds of Edman degradation.

### UVC and staurosporine-induced apoptosis

Cells were seeded into 10 cm dishes, allowed to attach overnight and exposed to indicated doses of UVC using a UVC crosslinker (Syngene GCL8S Crosslinker UV Radiation System Cleaver Scientific; Rugby, Warwickshire, UK used with a 254 nM wavelength) or treated with 1 *μ*M staurosporine. Live as well as necrotic and apoptotic floating cells were harvested following indicated time points.

### Subcellular fractionation

This was performed using the Thermo Scientific Subcellular Fractionation Kit (ThermoFisher; Brendale QLD, Australia), through a stepwise lysis and centrifugation of cells into functional cytoplasmic-, membrane-, nuclear-soluble, chromatin-bound and cytoskeleton protein fractions as per the manufacturer's instructions.

### NF-*κ*B inhibitor DHMEQ

The NF-*κ*B inhibitor DHMEQ from Kazuo Umezawa (Aichi Medical University, Nagakute, Japan) was added to cells 6 h following SASH1-GFP transfected in 96-well plates (200 ng DNA, 0.5 *μ*l Fugene HD per well). DHMEQ was added to the cells at 0, 1, 2, 5, 10, 20 or 50 *μ*g/ml. Cells were treated with DHMEQ for 48 h before cell death was assessed using Incell 2200 imaging and was analysed using Incell analysis software (GE HealthCare Lifesciences, Parramatta NSW, Australia), in combination with PI and Hoechst staining.

### Caspase inhibitor Z-VAD-FMK

Stably expressing ectopic SASH1-Flag U2OS cells were treated with UVC to induce cell death with the addition of Z-VAD-FMK (50 *μ*M) to inhibit caspase activity. Lysates were immunoprecipitated (1 mg) with M2-Flag beads (20 *μ*l) and SASH1 was eluted from the beads with 2 × SDS loading dye with 5% *β*-mercaptoethanol.

### Recombinant caspase-3 cleavage

SASH1 was immunoprecipitated from U2OS cells stably expressing ectopic SASH1. Beads were washed five times in IP buffer to remove contaminating proteins. SASH1-bound beads were incubated for 16 h in protease inhibitor-free buffer (25 mM HEPES, 0.1% (w/v) CHAPS, 10 mM DTT, pH 7.5) with and without 2 U of active recombinant caspase-3 (Abcam, Melbourne, VIC, Australia). SASH1 was then eluted from the beads with 2 × SDS loading dye containing 5% *β*-mercaptoethanol.

### Quantitative real-time PCR

Quantitative real-time PCR (qRT-PCR) was performed as described previously.^43^ In summary, 1 *μ*l diluted cDNA reverse transcribed from total RNA in nuclease-free H_2_O (1 : 5), 50 nM forward and reverse primer, 1x final concentration of SYBR Green PCR Master Mix (Applied Biosystems, ThermoFisher; Brendale QLD, Australia) and nuclease-free H_2_O (total volume of 10 *μ*l). Reactions were performed using a ViiA7 Real-Time PCR System (Life Technologies). Cycling conditions were 95 °C for 10 min, 40 cycles of 95 °C for 15 s and 60 °C for 1 min followed by a primer-template dissociation step. Gene expression was normalised to 7SL mRNA levels using the comparative CT (CT) method. The primer sequences for the human genes are given in Supplementary Table S1.

### Statistical analysis

Statistics analysis was performed using the GraphPad Prism analysis software (La Jolla, CA, USA). Student's *T-*test were performed with *P*-values as indicated, *P<0.05, **P<0.005 and ***P<0.0005.

## Abbreviations

Akt: protein kinase B
APAF1: apoptotic protease-activating factor 1
BAK: BCL2 antagonist/killer 1
BAX: BCL2-associated X protein
bcl-2: B-cell CLL/lymphoma 2
c-IAP1: cellular inhibitor of apoptosis protein-1
GFP: green fluorescent protein
IKK: I*κ*B kinase
NF-*κ*B: nuclear factor-*κ*B
PARP1: poly (ADP-ribose) polymerase
PVDF: polyvinylidene fluoride
PI3K: phosphatidylinositol-4,5-bisphosphate 3-kinase
SASH1: SAM and SH3 domain containing 1
siRNA: small interfering RNA
TAK1: transforming growth factor *β*-activated kinase 1
TRAF6: TNF receptor-associated factor 6, E3 ubiquitin-protein ligase
UVC: ultraviolet light C
XIAP: X-linked inhibitor of apoptosis protein
