# Supplementary material for: Activation and cleavage of SASH1 by caspase-3 mediates an apoptotic response
Source: Cell Death Dis. 2016 Nov 10;7(11):e2469–. doi: 10.1038/cddis.2016.364 (PMC5260870; doi:10.1038/cddis.2016.364)
Supplement: Supplementary Information [file cddis2016364x1.pdf]

Supplemental data "Activation and cleavage of SASH1 by Caspase-3 mediates an apoptotic response"

Joshua T. Burgess[1], Emma Bolderson[1,2*], Mark N. Adams[1], Anne-Marie Baird[1], Shu-Dong Zhang[3,4], Kathy A. Gately[5], Kazuo Umezawa[6], Kenneth J. O'Byrne[1,2], and Derek J. Richard[1,2*].

1. Cancer & Ageing Research Program, Institute of Health and Biomedical Innovation at the Translational Research Institute (TRI), Queensland University of Technology (QUT), Brisbane, Australia.
2. Princess Alexandra Hospital, Ipswich Road, Woolloongabba, Brisbane, Queensland 4102, Australia.
3. Northern Ireland Centre for Stratified Medicine, University of Ulster, C-TRIC Building, Altnagelvin Hospital campus, Glenshane Road, Londonderry, BT47 6SB, UK.
4. Center for Cancer Research and Cell Biology, Queen's University Belfast, United Kingdom.
5. Thoracic Oncology Research Group, Institute of Molecular Medicine, Trinity College Dublin, St. James's Hospital, Dublin, Republic of Ireland
6. Department of Molecular Target Medicine Screening, Aichi Medical University, Nagakute, Japan.

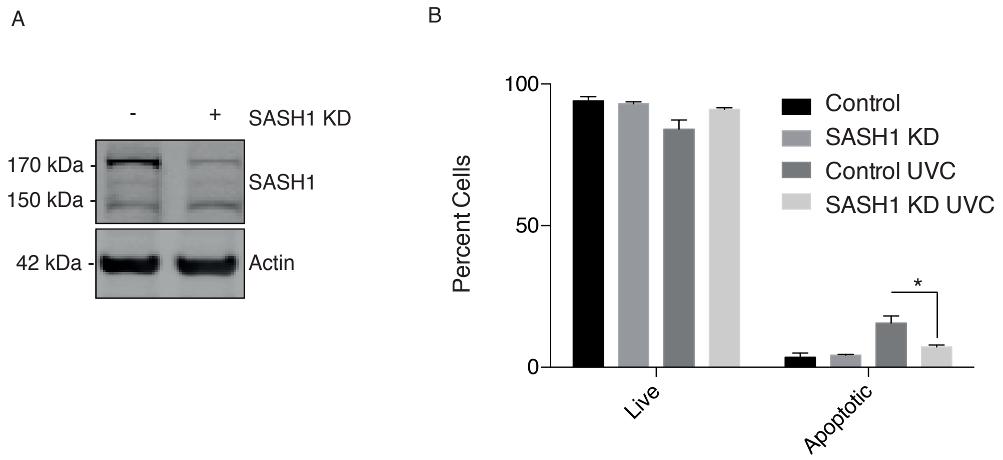

Supplemental Figure 1: Depletion of SASH1 in A549 cells increases resistance to UVC-induced apoptosis. A) A549 cells were transfected with SASH1 or control esiRNA. 72 hours after transfection cell lysates were immunoblotted with SASH1 and β-actin antibodies. B) A549 cells were transfected with control or SASH1 siRNA. 72 hours after transfection cells were treated with UVC (30mJ/cm^2) and harvested after 3 h. Cells were stained with Annexin V 488 and propidium iodide and analysed on Gallios Flow cytometer. The percentage of live and apoptotic cells is shown. Data shown are the means +/- standard deviation from three independent experiments. Statistical analysis performed with student T test with * $P < 0.01$.

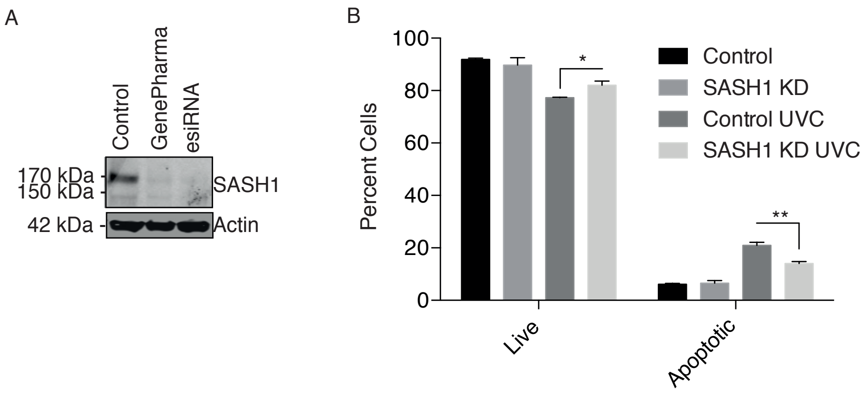

Supplemental Figure 2: SASH1 depletion with GenePharma siRNA inhibits apoptosis. A) HeLa cells were transfected with SASH1 or control siRNA. 72 hours after transfection cell lysates were immunoblotted with SASH1 and β-actin antibodies. B) HeLa cells were transfected with control or SASH1 siRNA from GenePharma. 72 hours after transfection cells were treated with UVC (30mJ/cm2) and harvested after 3 h. Cells were stained with Annexin V 488 and Propidium iodine and analysed by Flow cytometry, using a Gallios flow cytometer (Beckman) and quantified using the Kaluza software (Beckman). The percentage of live and apoptotic cells is shown. Data shown are the means +/- standard deviation from three independent experiments. Statistical analysis performed with student T test with ** P < 0.001, * P < 0.01.

A

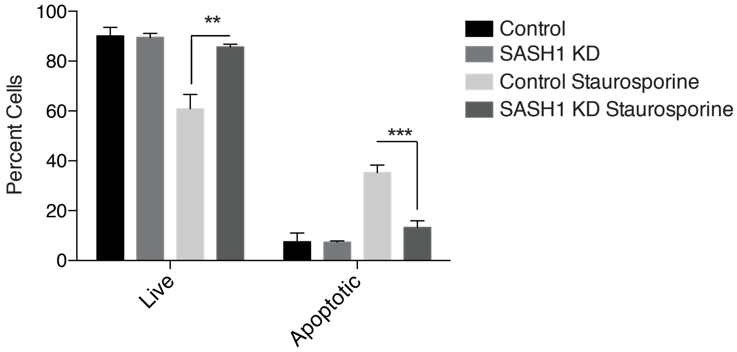

Supplemental Figure 3: SASH1 depleted cells are resistant to staurosporine-induced apoptosis. A) HeLa cells were transfected with control or SASH1 esiRNA. 72 hours after transfection cells were treated with staurosporine (1 μM) and harvested after 6 h. Cells were stained with Annexin V 488 and Propidium iodide and analysed via Flow cytometry using a Gallios flow cytometer (Beckman) and quantified using the Kaluza software (Beckman). The percentage of live and apoptotic cells is shown. Data shown are the means +/- standard deviation from three independent experiments. Statistical analysis performed with student T test with ** $P < 0.001$, *** $P < 0.0001$.

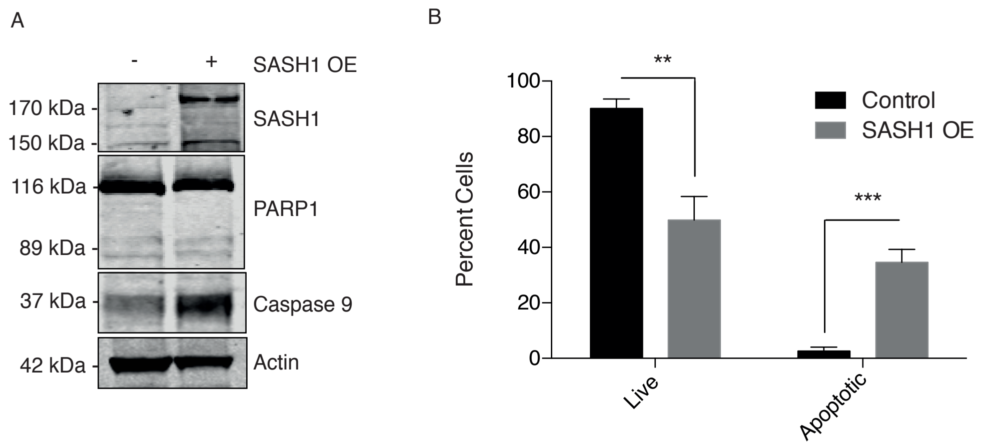

Supplemental Figure 4: SASH1 overexpression in A549 cells induces apoptosis. A) A549 cells were transfected with SASH1 WT. 48 hours after transfection cell lysates were harvested and immunoblotted with SASH1, Caspase-9, PARP1 and β-actin antibodies. B) Annexin V staining of A549 cells 48 hr following overexpression of control (empty PCMV6 vector) or SASH1 WT. Flow cytometry was performed using a Gallios flow cytometer (Beckman) and quantified using the Kaluza software (Beckman). The percentage of live and apoptotic cells are shown. Data shown are the means +/- standard deviation from three independent experiments. Statistical analysis were performed with student T test with ** $P < 0.001$, *** $P < 0.0001$.

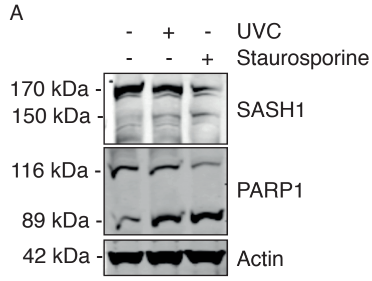

Supplemental Figure 5: SASH1 is cleaved in response to staurosporine-induced apoptosis. A) Immunoblot of UVC (6 hr post 30 mj/cm^2) or staurosporine-treated HeLa cell lysates (6 h post 1 μM) indicating cleavage of SASH1 from 170 kDa to 150 kDa. PARP1 antibodies were also used as a marker of apoptosis.

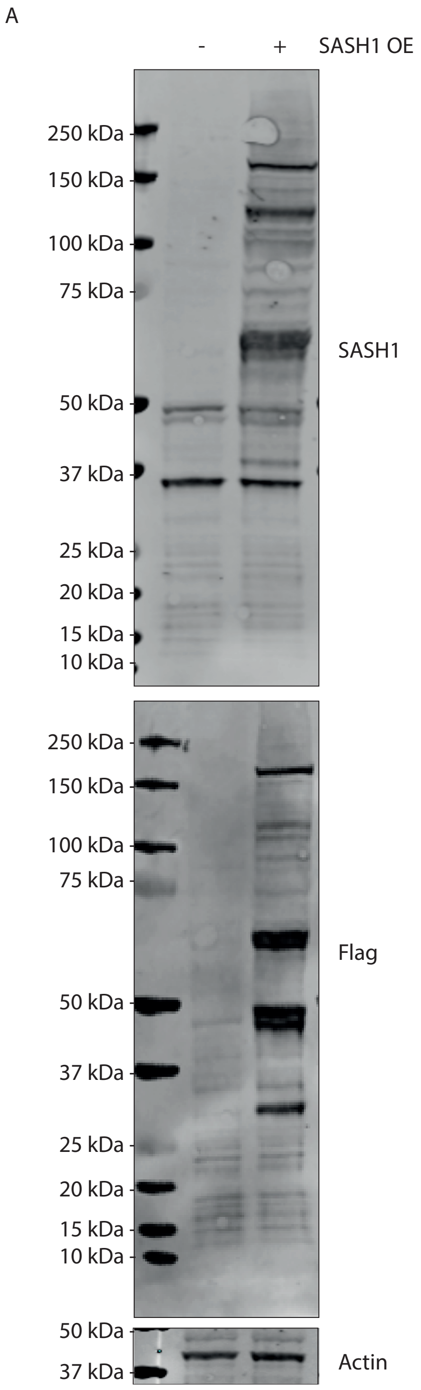

Supplemental Figure 6: Immunoblot demonstrating SASH1 antibody specificity. A) Immunoblot of U2OS cell lysates following overexpression of Flag-SASH1 (24 hours) incubated with SASH1 or Flag antibodies, with molecular weight marker indicated.

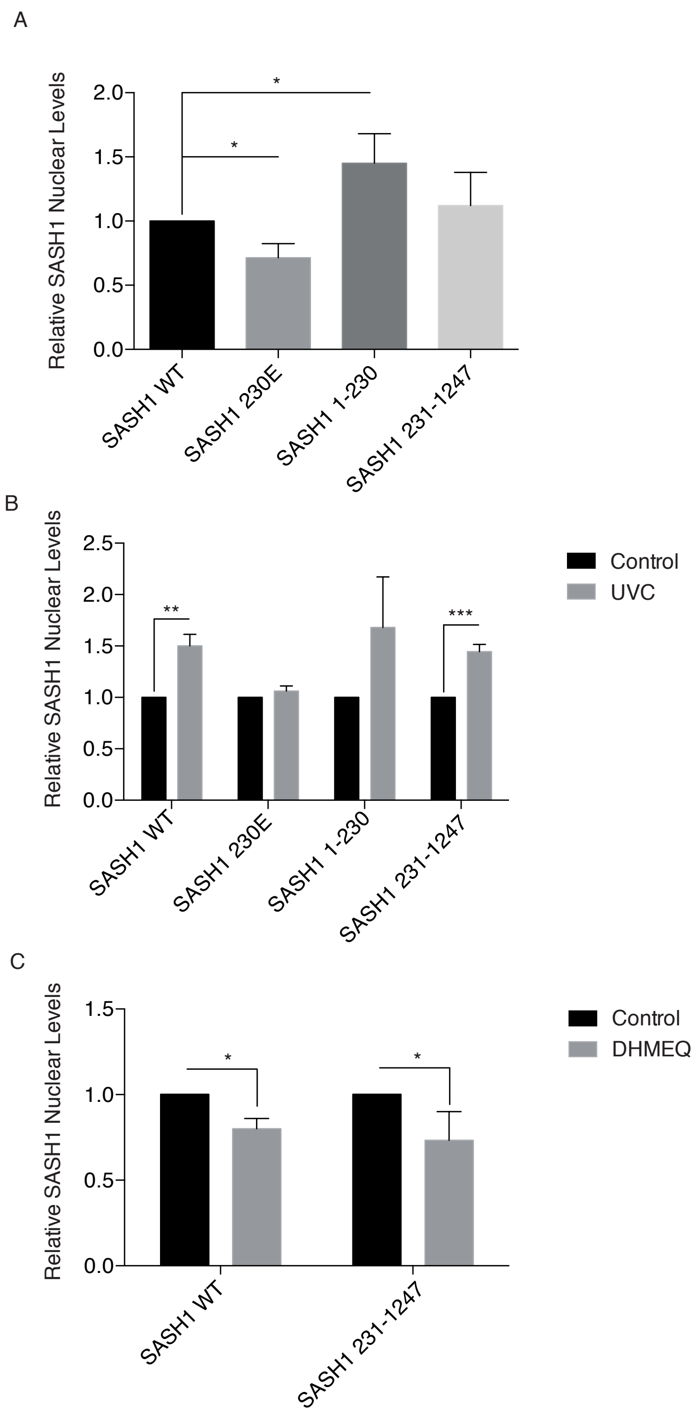

Supplemental Figure 7: SASH1 cleavage and NF-κB activation is required for SASH1 nuclear localisation. A) Nuclear levels of MYC-tagged overexpressed SASH1 relative to cytoplasmic levels. Significantly less nuclear 230E uncleavable SASH1 was observed when compared to wildtype SASH1. B) Relative nuclear levels

of SASH1 following UVC induced apoptosis (3 hr, 30 mj/cm^2). SASH1 wild type and 231-1247 show a significant increase in relative SASH1 nuclear levels to cytoplasmic levels following UVC treatment. C) Relative nuclear to cytoplasmic levels of SASH1 are significantly reduced in the presence of the NF-κB inhibitor DHMEQ. Cells were transfected with SASH WT or SASH1 231-1247 then left for 6 h before the addition of DHMEQ 10 μg/ml. A-C) HeLa cells were fixed 8 hr following transfection and Immunofluorescence was performed using MYC antibody. Quantification and statistical analysis was performed with InCell 2200 and InCell analysis software. The data represents the average and standard deviation of 3 independent experiments. Unpaired T test (* $P < 0.05$, ** $P < 0.005$, *** $P < 0.0005$).

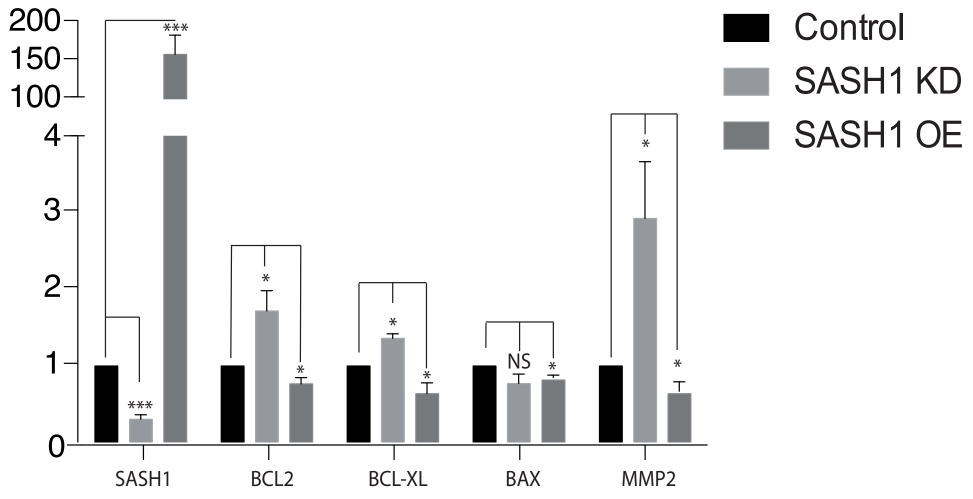

Supplemental Figure 8: SASH1 depletion or overexpression significantly alters the expression of NF-κB regulated genes. Real-time RT-PCR analysis of SASH1, BCL2, BCL-XL, MMP2 and BAX mRNA expression in HeLa cells treated with or without esiRNA or overexpression of SASH1. Bar graph representing the fold changes of mRNA levels quantitated by normalization to the 7SL as an internal control. Statistical analysis were performed with student T test with * $P < 0.01$, *** $P < 0.0001$. Mean values ± SD (n = 3). Depletion of SASH1 resulted in a significant increase in anti-apoptotic genes (BCL2, BCL-XL and MMP2) mRNA levels whilst the overexpression resulted in a significant decrease.

Supplementary Table 1:

**Oligo name**	**DNA sequence (5' to3')**
7SL forward	ATCGGGTGTCCGCACTAAGTT
7SL reverse	CAGCACGGGAGTTTTGACCT
SASH1 forward	AATTGAGGAAGCACTTGCTAGG
SASH1 reverse	ACCATCTGGCCAGTCAGC
BCL2 forward	CTGCACCTGACGCCCTTCACC
BCL2 Reverse	CACATGACCCCACCGAACTCAAAGA
BCL-X forward	GATCCCCATGGCAGCAGTAAAGCAAG
BCL-X reverse	CCCCATCCCGGAAGAGTTCATTCACT
MMP2 Forward	TCTCCTGACATTGACCTTGGC
MMP2 Reverse	CAAGGTGCTGGCTGAGTAGATC
BAX Forward	CATGTTTTCTGACGGCAACTTC
BAX reverse	AGGGCCTTGAGCACCAGTTT
